# BK Polyomavirus-Induced Nephropathy in Native Kidney

**DOI:** 10.7759/cureus.34410

**Published:** 2023-01-30

**Authors:** Ripudaman S Munjal, Jaskaran Munjal, Pulkit Gandhi, Nikhil Gupta

**Affiliations:** 1 Nephrology, San Joaquin General Hospital, Stockton, USA; 2 Internal Medicine, Shri Ram Murti Smarak Institute of Medical Sciences, Bareilly, IND; 3 Nephrology, Lake Erie College of Osteopathic Medicine, Erie, USA; 4 Nephrology, Rochester Regional Health, Rochester, USA; 5 Internal Medicine, San Joaquin General Hospital, Stockton, USA

**Keywords:** nephropathy, bk polyomavirus, immunosuppressed, dialysis, chronic kidney disease, acute kidney injury, bk nephropathy

## Abstract

BK polyomavirus has been well-studied as an opportunistic infection in immunocompromised kidney transplant patients. In the majority of the population, BK polyomavirus establishes a lifelong infection in renal tubular and uroepithelial cells; however, in an immunocompromised state, the virus can reactivate and can lead to BK polyomavirus-associated nephropathy (BKN). In this case, the patient was a 46-year-old male with a past medical history of HIV, compliant with antiretroviral therapy (ART), and B-cell lymphoma treated with chemotherapy. The patient presented with worsening kidney function of unknown etiology. This prompted further assessment with a kidney biopsy. Kidney biopsy findings were consistent with BKN. In the literature, BKN has been studied in renal transplant patients; however, it rarely involves native kidneys.

## Introduction

The BK virus is a human polyomavirus that was first discovered in 1971 in the urine of a kidney transplant recipient [1]. It shares similar features with the simian virus 40 (SV40) and JC virus. Infection with the BK virus is endemic; the primary BK virus is typically acquired in childhood, with the seroprevalence rate in adults reaching 80% [2,3]. After infection, the virus remains dormant within the genitourinary epithelium. It is usually asymptomatic in immunocompetent hosts [4]. BK polyomavirus can cause BK nephropathy (BKN), ureteral stricture, and hemorrhagic cystitis in immunocompromised hosts [5]. BKN was thought to be a disease of kidney allografts, only affecting kidney transplant recipients. BKN occurs in 1% to 10% of patients with kidney transplantation [6]. Native kidney BKN is rare, especially without a history of prior organ transplantation. There is growing literature on BKN in immunocompromised patients [7]. Definitive diagnosis is made by a kidney biopsy and detection of polyomavirus in tubular cells of the kidney with the antigen simian virus 40 (SV40) of the virus using the SV40 stain [8]. Here, we describe a biopsy-proven native kidney BKN in an immunocompromised patient with no history of organ transplantation.

## Case presentation

A 46-year-old male was admitted to the hospital with pain in the abdomen and an acute kidney injury. His medical history included HIV, diagnosed about two years before presentation, and B-cell lymphoma, diagnosed about one year before presentation. B-cell lymphoma was treated with rituximab, etoposide phosphate, prednisone, vincristine sulfate, cyclophosphamide, and doxorubicin hydrochloride (R-EPOCH). B-cell lymphoma was in remission, and no further treatment was planned. HIV was treated with a combination pill containing bictegravir, emtricitabine, and tenofovir alafenamide. Despite the patient being compliant with his HIV medications, his clusters of differentiation 4 (CD4) count was low (25 cells/mm3). The HIV viral load was not checked at the time of presentation. The patient did not have any other complaints other than a pain in the abdomen, which resolved spontaneously.

He was noted to have had progressive worsening of kidney function over the course of six months prior to presentation. Creatinine worsened from 1.0 mg/dL with an eGFR of 72 mL/min/1.73m2 to creatinine of 3.63 mg/dL with an estimated glomerular filtration rate (eGFR) of 18 mL/min/1.73m2 [Table 1]. Urinalysis with urine microscopy was unremarkable. The kidney ultrasound was unremarkable. The cause of the progressive worsening of kidney function was unknown, prompting a kidney biopsy. Kidney biopsy results confirmed BKN with a positive stain for SV40. Serum and urine samples were also checked for BK polyomavirus. Serum BK virus polymerase chain reaction (PCR) showed 5,391,435 copies/mL (normal: <500 copies/mL), and urine BK virus PCR showed > 500 million copies/mL (normal: <500 copies/mL).

**Table 1:** Kidney function tests of the patient Serum creatinine is measured as milligrams per deciliter (mg/dL); eGFR: estimated glomerular filtration rate; eGFR is measured as milliliters per minute per body surface (mL/min/1.73m2)

Date	Serum creatinine (mg/dL)	eGFR (mL/min/1.73m2)
August 2021	1	72
September 2021	1.95	37
October 2021	2.25	34
February 2022	3.63	18

The patient could not receive any of the treatment options. The patient was followed up after hospital discharge but was noted to have continued worsening kidney functions. He unfortunately required kidney replacement therapy in the form of hemodialysis four months after the initial presentation.

Pathology 

**Figure 1:**
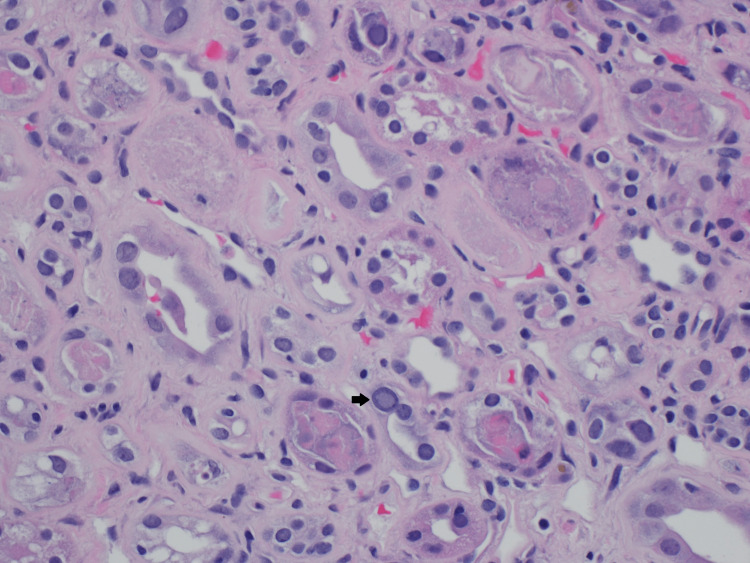
Kidney biopsy with viral inclusions

**Figure 2:**
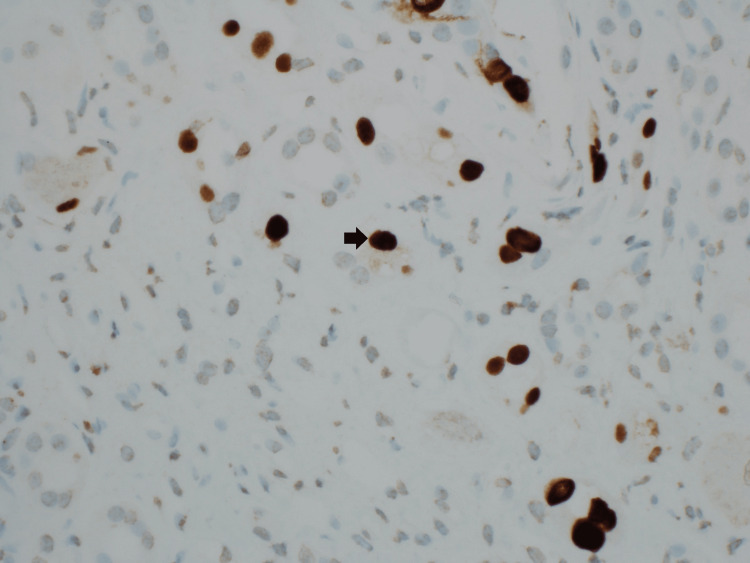
Kidney biopsy: simian virus 40 (SV40) immunostain

The sample for pathology contained a small core. One of the five glomeruli was globally sclerotic. Glomeruli were histologically unremarkable. Numerous viral inclusions (Figure 1) involving a large number of cells were seen. SV40 immunostain (Figure 2) was positive in nuclei with inclusions indicative of polyomavirus nephropathy. Immunofluorescence had no specific staining of immunoglobulin G (IgG), immunoglobulin A (IgA), immunoglobulin M (IgM), complement component 3 (C3), kappa, or lambda light chains.

## Discussion

The BK virus is a DNA virus of the Polyomaviridae family that causes interstitial nephritis in immunosuppressed patients [9]. It is caused by the reactivation of the BK virus, which leads to inflammation and damage to the kidney tissue. It is a known occurrence after kidney transplantation. BKN usually affects 1%-15% of kidney transplant recipients, which can be associated with allograft loss [10,11]. The BK virus is rare in native kidneys; most cases have been reported in bone marrow transplant patients [7]. There is limited literature on non-transplant native kidney BKN. BKN is a potential cause of kidney dysfunction in both native and transplanted kidneys. A high percentage of patients (21.5%) who are diagnosed with BKN require chronic dialysis, as in the case report presented here [12]. This makes it very important to consider the BK virus as a potential cause of worsening kidney functions, especially in immunosuppressed individuals. More cases being reported are helping to better understand the disease.

The diagnosis of BKN includes BK virus PCR in blood and urine, but a definitive diagnosis is made by a kidney biopsy [8]. The non-invasive method of initial diagnostic tests may be readily available and help in early diagnosis. The utility of screening immunocompromised patients with PCR remains to be studied.

No effective therapy is currently available for BKN. In kidney transplant recipients, a reduction in immunosuppression can be effective in treating BKN [4]. In individuals where reduction in immunosuppression is not possible or in immunocompetent patients, leflunomide can be considered, as it has shown some benefits [13,14,15]. Other alternate therapies include rapamycin (mTOR) inhibitors, fluoroquinolones, cidofovir, and intravenous immunoglobulin (IVIg) [16,17,18,19].

In the case we present, the patient was not able to get any of the available treatment options. The HIV medications were continued. Although the HIV viral load was not checked initially, it was undetectable on the follow-up visit, and the CD4 count continued to be low. Unfortunately, the patient's kidney function continued to worsen, requiring kidney replacement therapy.

## Conclusions

BKN should be considered in the differential diagnosis for worsening kidney function in immunocompromised patients with no clear diagnosis, even in patients who are not organ transplant recipients. Non-invasive tests can be considered for possible early diagnosis. As it has a high incidence of progressing to end-stage kidney disease requiring chronic kidney replacement therapy, early diagnosis and prompt treatment can prove to be beneficial.
